# Possible psychotic episode after repeated ayahuasca intake: a case report

**DOI:** 10.1192/j.eurpsy.2023.1584

**Published:** 2023-07-19

**Authors:** E. Prades Marin, A. Martínez Torres, P. Sánchez Díez

**Affiliations:** Psychiatry, Hospital Universitario Ramón y Cajal, Madrid, Spain

## Abstract

**Introduction:**

Ayahuasca is a traditional brew containing the psychedelic 5-DMT (a tryptamine that acts as a 5HT2A-R agonist) used to achieve non-ordinary states of consciousness, with a long tradition among various cultures in ritual and therapeutic contexts. Ayahuasca is being studied as potential treatment in Mental Health, which has led to non-controlled and recreational use. This has led to a rise in the description of side effects, such as substance-induced psychosis.

**Objectives:**

To describe a case of a possible psychotic episode related to the intake of ayahuasca brew in a ritual context.

**Methods:**

Clinical assesment and bibliographic review of pertinent literature.

**Results:**

We will present the case of a 43 year old woman, who participated in three ayahuasca sessions in three consecutive months. Two days after the last session, she suffered and episode of loss of consciousness, convulsions, loss of streng and paraesthesia in right forearm and righr side of the face and head; and apparition of perceptual alterations and delusions that she did not experience during the trip. Such alterations included the perception of electromagnetic fields (EMF) robbing her of her vital energy and lifeforce. She required ICU treatment for four days, after which se was hospitalized in Internal Medicine Unit and was assessed by Mental Health team. Though the symptoms were coherent with the previous beliefs of the patient, they were clearly exacerbated and interfered with her normal and previous functioning. She was treated with risperidone 1,5 mg, with complete symptom remission.

**Conclusions:**

The case presented is consistent with other reports of ayahuasca-induced psychotic symptoms, though with less intensity and duration. We discuss prevalence and repercussions of the rising use of this powerful substance; that must be taken into consideration by clinicians worldwide.

**Disclosure of Interest:**

None Declared

